# The Distribution of Lifestyle Risk Factors Among Patients with Stroke in the Indian Setting: Systematic Review and Meta-Analysis

**DOI:** 10.1177/09727531221115899

**Published:** 2022-08-08

**Authors:** Biji P. Varkey, Jaison Joseph, Abin Varghese, Suresh K. Sharma, Elezebeth Mathews, Manju Dhandapani, Venkata Lakshmi Narasimha, Radha Kuttan, Saleena Shah, Surekha Dabla, Sivashanmugam Dhandapani

**Affiliations:** 1 Department of Neurology, Postgraduate Institute of Medical Sciences, Pandit Bhagwat Dayal Sharma University of Health Sciences, Rohtak, Haryana, India; 2 Department of Psychiatric Nursing, College of Nursing, Pandit Bhagwat Dayal Sharma University of Health Sciences, Rohtak, Haryana, India; 3 College of Nursing, AIIMS, Nagpur, India; 4 College of Nursing, All India Institute of Medical Sciences (AIIMS), Jodhpur, Rajasthan, India; 5 Department of Public Health and Community Medicine, Central University of Kerala, Kasaragod, Kerala, India; 6 National Institute of Nursing Education, Postgraduate Institute of Medical Education and Research (PGIMER), Chandigarh, Chandigarh, India; 7 Department of Psychiatry, All India Institute of Medical Sciences (AIIMS) Deoghar, Jharkhand, India; 8 College of Nursing, Bhopal Memorial Hospital and Research Centre, ICMR, Bhopal, Madhya Pradesh, India; 9 Government College of Nursing Thiruvananthapuram, Kerala, India; 10 Department of Neurology, Pandit Bhagwat Dayal Sharma University of Health Sciences, Rohtak, Haryana, India; 11 Department of Neurosurgery, Postgraduate Institute of Medical Education and Research (PGIMER), Chandigarh, Chandigarh, India

**Keywords:** Stroke, Lifestyle, Risk factors, Patients, India

## Abstract

**Background:**

The burden of stroke is increasing in India, but there is limited understanding of the distribution of reported risk factors in the Indian setting. It is vital to generate robust data on these modifiable risk factors to scale up appropriate strategies for the prevention of cerebrovascular diseases in this setting.

**Summary:**

The objective of this study is to estimate the overall proportion of life style risk factors of patients with stroke in the Indian setting. We searched PubMed and Google Scholar and relevant studies published till February 2022 were included. The risk of bias assessment was considered for the study selection criterion in the meta-analysis. The publication bias was evaluated by funnel plots and Egger’s test. We identified 61 studies in the systematic review and after quality assessment, 36 studies were included for meta-analysis. Random effect model was used due to the significant inconsistency among the included studies (I2 > 97%). The mean age of the participants was 53.84±9.3 years and patients with stroke were predominantly males (64%). Hypertension (56.69%; 95% CI: - 48.45 – 64.58), obesity (36.61%; 95% CI: - 19.31 – 58.23), dyslipidemia (30.6%; 95% CI: - 22 – 40.81) and diabetes mellitus (23.8%; 95% CI: - 18.79 – 29.83) are the leading intermediate conditions associated with stroke. The Physical inactivity - 29.9% (95% CI: - 22.9 – 37.1), history of tobacco use (28.59 %; 95% CI: - 22.22 – 32.94) and alcohol use (28.15 %; 95% CI: - 20.49 – 37.33) were reported as the behavioral risk factors for stroke in this setting.

**Key Messages:**

The current meta-analysis provides robust estimates of the life style related risk-factor of stroke in India based on the observational studies conducted from 1994 to 2019. Estimating the pooled analysis of stroke risk factors is crucial to predict the imposed burden of the illness and ascertain the treatment and prevention strategies for controlling the modifiable risk factors in this setting.

## Introduction

Cardiovascular diseases accounted for the majority of deaths globally [18·6 million (17·1–19·7)] in both sexes combined in 2019, amongst which stroke is the second leading cause of death, 3·33 million (3·04–3·62) stroke deaths in males and 3·22 million (2·86–3·54) deaths in females.^1^ Disability-adjusted life years (DALYs) for cardiovascular diseases were 393 million (95% UI 368–417) and 143 million (95% UI 133–153) for stroke, making stroke the third-leading cause of disease burden.^2^ In India, cardiovascular diseases attributed the highest percent of total death for all ages in which stroke was the fifth leading cause of death in the year 2016, with a mean percent change in the number of DALYs of 52.9% (40.4–66.7) between 1990 and 2016.^3^ Within India, a wide variation in the burden of stroke was observed across the states. To cite, a recent meta-analysis reported a one-month case fatality rate of stroke varied from 41.08% to 42.06% in the urban population and 18% to 46.3%.in the rural population.^4^ This wide variation could be because of the variability in the distribution of risk factors in the population, effectiveness of health services in preventive, curative, and rehabilitative services, and data availability.^5^

The available empirical data reported the risk factors for stroke, such as sociodemographic, behavioral, anthropometric, clinical, and biochemical, from multiple settings in India.^6, 7^ The significant lifestyle-related risk factors include hypertension, diabetes, hyperlipidemia, obesity, smoking, heart disorders, congestive cardiac failure, atrial fibrillation, left ventricular hypertrophy), and so on, and the burden of each risk factor remains unknown.^8^ The global burden of disease study (1990–2016) reported a gross variation in the risk factors for cardiovascular disease across the states of India.^9^ It is also evident that South Asians, including Indians, are highly vulnerable to cardiovascular disease because of their cardiometabolic risk profile and ethnically mediated cardiometabolic dysfunction.^10^

The burden of stroke is increasing in India, but there is scanty evidence on the systematic understanding of the distribution of its lifestyle risk factors in the Indian setting. It is vital to generate robust data on these risk factors to scale-up appropriate strategies for preventing cerebrovascular diseases in this setting.

## Objective

The objective of this study was to estimate the overall proportion of lifestyle risk factors of patients with stroke in the Indian setting.

## Materials and Methods

### Search Strategy and Selection Criteria

This systematic review is reported following the PRISMA checklist.^11^ We searched PubMed and Google Scholar, and relevant studies published till February 2022 were included. We used the combination of Medical Subject Headings (MeSH) and keywords of the following search concepts: “stroke,” “risk factors,” “patients,” and “India.” The details of the search strategy in PubMed are given as supplementary material 1. The data search was carried out by two investigators (BPV and MD). The archives of relevant Indian journals were reviewed for maximum inclusion of available studies. No attempts are made to acquire grey/unpublished literature considering the inherent conflict of interest, which might increase the risk of bias. The screening was performed by two investigators (RK and SS) who further appraised the full texts of appropriated records to reach a common consensus regarding the inclusion and exclusion of individual studies.

### Inclusion and Exclusion Criteria

Observational studies, both hospital and community-based stroke registry studies, conducted in the Indian setting reporting the risk factors of various types of strokes and published in the English language were included. Stroke registries are observational databases focusing on the clinical information and outcomes of stroke patients. Stroke is a chronic disease with an acute event, so the hospitalization rate is high. As we were not estimating any incidence or prevalence of stroke, we also included hospital-based clinical studies recognizing its limitations and inherent biases.

Studies were included if participants had a confirmed history of stroke as defined by the World Health Organization (WHO)^12^ or as defined according to clinical criteria or confirmed by imaging. Global or Indian studies that exclusively estimated the prevalence, incidence, and mortality data among patients with stroke were excluded. Besides, studies with inadequate data, published as editorials or letters to the editor, conference abstracts, expert opinion, or suggestions were excluded. The lifestyle risk factors for stroke were operationally defined as the conditions and behaviors that increases the chances of an individual to have, develop, or be adversely affected by a disease process. In this study, this was categorized into behavioral risk factors and intermediate conditions. The data on the nonmodifiable risk factors of stroke, such as age, previous history of stroke, and family history of stroke, were not estimated.

### Data Extraction

The data extraction was done based on the following study characteristics: author (year of publication)/study region, period of study (year), types of stroke, mean age, gender, sample size, and risk factors (hypertension, diabetes mellitus, tobacco use, alcohol use, dyslipidemia, and others). Three investigators (BPV, JJ, and AV) were involved in the data extraction after reading and discussing the full-text version of the shortlisted publications based on the eligibility criteria. The extracted data were cross-verified by the author VLN and SD. A mutual consensus resolved disagreements between the authors (BV, JJ, AV, VLN, and SD).

### Quality Assessment

The Joanna Briggs Institute (JBI) critical appraisal checklist was used for the risk of bias assessment (available from https://synthesismanual.jbi.global). The assessment of each study was done based on whether it met the following eight conditions: (a) Were the criteria for inclusion in the sample clearly defined? (b) Were the study subjects and the setting described in detail? (c) Was the exposure measured validly and reliably? (d) Were objective and standard criteria used to measure the condition? (e) Were confounding factors identified? (f) Were strategies to deal with confounding factors stated? (g) Were the outcomes measured validly and reliably? (h) Was appropriate statistical analysis used? Each study was graded as 1–Yes; 0–No; UC–Unclear after rigorous review. The total scores of the included studies were considered for the study selection criterion in the meta-analysis. Three review authors independently assessed the risk of bias in the included studies (SKS, EM, and JJ). This process was performed iteratively. First, each author reviewed the studies and made the risk of bias assessment based on the criteria. A third independent reviewer (SSD) addressed discrepancies in the quality scoring of two reviewers. Disagreements were resolved by group consensus.

### Statistical Analysis

The R software was used to perform this meta-analysis, and the pooled estimate of the distribution of lifestyle risk factors of stroke was estimated using inverse variance weighting methods. Assuming the significant inconsistency among the studies, a random-effects meta-analysis model was used, and *I*^2^ statistics were calculated to measure heterogeneity among studies. The heterogeneity was considered mild, moderate, or high when the *I*^2^ values were from 25% to 50%, 51% to 75%, and >75%, respectively. The funnel plot and Egger’s test were used to assess the potential publication bias.

## Results

### Identification of Studies

**Figure 1.**
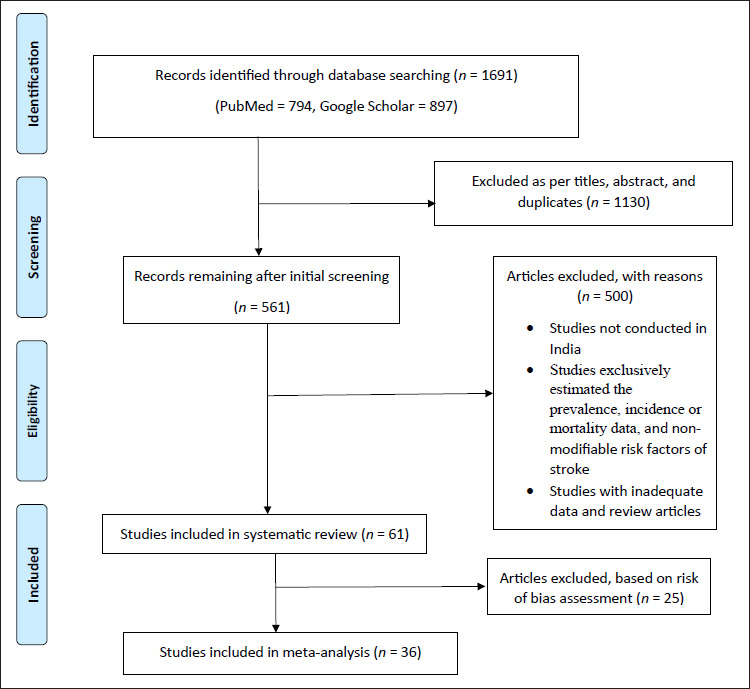
Flowchart of Search Strategy and Selection Process.

The database search identified 1691 reports: 1130 were excluded based on title and abstract screening, and after eliminating duplicates, 561 articles were retrieved for detailed evaluation, and 500 were excluded for the reasons summarized in Figure 1. Finally, 61 eligible articles were identified after applying the inclusion and exclusion criteria and included in the systematic review, out of which 36 were included in the meta-analysis based on the risk of bias assessment.

### Characteristics of the Studies Included in the Systematic Review

We have included 61 studies in our systematic review, conducted across various states in India, estimating the various lifestyle risk factors for stroke (Table 1).^13–73^ Out of 61, 56 studies were conducted and 58 were published after 2000. In 51 studies, patients were enrolled from the hospital-based stroke registries (HBSR) maintained in various treatment settings across India, while in 10 studies, the enrollment was using population-based stroke registries (PBSR). The sample size of included studies varied from 32 to 4989. The number of patients with ischemic stroke and hemorrhagic stroke in the included studies ranged from 19 to 3260 and 3 to 1656, respectively. Other types of strokes reported in a few studies varied from 8 to 271.

**Table 1.** Characteristics of the Included Studies in the Systematic Review (*N* = 61).

Author (Year of Publication)/Study Region	Period of Study (Year)	Study Type	Sample Size	Type of stroke			JBI Score
				Ischemic	Hemorrhagic	Other	
Singla et al. (2022)/Punjab^13^	2010–2013	PBSR	2948	1890	787	271	7
Jayadevappa and Ravishankar (2021)/Karnataka^14^	2013–2014	HBSR	230	200	30	NA	5
Ram et al. (2021)/Across India^15^	2016–2017	HBSR	526	299	98	129	4
Rathore et al. (2021)/Rajastan^16^	2019	HBSR	100	72	28	NA	4
Kumar et al. (2021)/Uttarakhand^17^	2018–2019	HBSR	48	39	9	NA	5
Ahmed et al. (2020)/Uttarakhand^18^	2019–2020	HBSR	129	122	07	NA	3
Prabhakar et al. (2020)/Telengana^19^	2016–2017	PBSR	144	NM	NM	NM	8
Kaur et al. (2020)/Rajasthan^20^	2015–2016	HBSR	360	290	70	NA	5
Somasundaran and Potty (2020)/Kerala^21^	2014–2015	HBSR	464	335	129	NA	3
Moond et al. (2020)/New Delhi^22^	2014–18	HBSR	160	160	NA	NA	3
Karri and Ramasamy (2019)/Tamil Nadu^23^	2014–2017	HBSR	186	186	NA	NA	5
Patel and Vagadiya (2019)/Gujarat^24^	2014	HBSR	46	46	NA	NA	4
Panwar et al. (2019)/Madhya Pradesh^25^	2013–2014	HBSR	50	39	11	NA	5
Muralidharan et al. (2019)/Kerala^26^	2018–2019	HBSR	200	173	27	NA	4
Rajan et al. (2019)/Karnataka^27^	2013–2014	PBSR	150	52	05	93	4
Behera and Mohanty (2019)/Odisha^28^	2018–2019	HBSR	796	481	315	NA	5
Pathak et al. (2018)/New Delhi^29^	2012–2014	HBSR	268	169	60	39	5
Hussain et al. (2018)/Meghalaya^30^	2016–2017	HBSR	150	76	62	12	3
Sylaja et al. (2018)/Across India^31^	2012–2014	HBSR	2066	NM	NM	NM	5
Diwan et al. (2018)/Maharashtra^32^	2016–2017	HBSR	70	35	35	NA	3
Kaur et al. (2017)/Punjab^33^	2011–2013	PBSR	4989	3260	1656	47	8
Kabi et al. (2017)/Odisha^34^	2014–2016	HBSR	367	218	149	NA	3
Chandran et al. (2017)/Kerala^35^	2010	PBSR	40	37	03	NA	3
Mahanta et al. (2018)/Assam^36^	2013–2015	HBSR	450	163	287	NA	7
Chandana and Kalyani (2017)/Andhra Pradesh^37^	2016–2017	HBSR	50	30	11	9	4
Jacob and Kulkarni (2017)/Karnataka^38^	2012–2013	PBSR	53	NM	NM	NM	3
Kumar and Rai (2017)/Uttar Pradesh^39^	NM	HBSR	100	NM	NM	NM	5
Manorenj et al. (2016)/Telengana^40^	2015–2016	HBSR	100	76	24	NA	7
Nayak et al. (2016)/Madhya Pradesh^41^	2011–2013	HBSR	104	104	NA	NA	6
Huliyappa and Kotrabasappa (2016)/Karnataka^42^	2013–2014	HBSR	52	NM	NM	NA	5
Khan et al. (2015)/Chhattisgarh^43^	2014	HBSR	281	190	91	NA	2
Jadhav and Bondarde (2015)/Maharashtra^44^	2011–2013	HBSR	40	22	10	8	4
Subha et al. (2015)/Kerala^45^	2013	HBSR	100	71	29	NA	8
Vaidya et al. (2015)/Gujarat^46^	2012–2013	HBSR	175	175	NA	NA	4
Kawle et al. (2015)/Maharashtra^47^	2012–2014	HBSR	104	104	NA	NA	6
Shravani et al. (2015)/Karnataka^48^	2010–2011	HBSR	100	74	26	NA	7
Renjen et al. (2015)/New Delhi^49^	2004–2006	HBSR	244	244	NA	NA	7
Jan et al. (2015)/Jammu & Kashmir^50^	2011	HBSR	209	NM	NM	NA	4
Gupta et al. (2014)/Chandigarh^51^	NM	HBSR	73	73	NA	NA	5
Kapoor et al. (2014)/Himachal Pradesh^52^	2012–2013	HBSR	32	19	13	NA	3
Sorganvi et al. (2014)/Karnataka^53^	NM	HBSR	100	NM	NM	NM	8
Dash et al. (2014)/New Delhi^54^	2005–2010	HBSR	440	440	NA	NA	6
Kulshrestha and Vidyanand (2013)/Uttar Pradesh^55^	2011–2012	HBSR	157	112	45	NA	5
Singh et al. (2013)/Punjab^56^	2006–2011	HBSR	1156	838	318	NA	8
Deoke et al. (2012)/Maharashtra^57^	NM	HBSR	101	86	15	NA	8
Narayan et al. (2012)/Telengana^58^	2002–2010	HBSR	428	NA	NA	428	4
Kumar et al. (2011)/Karnataka^59^	1998–2008	HBSR	109	84	25	NA	6
Raju et al. (2010)/Punjab^60^	2008–2010	HBSR	162	125	37	NA	3
Kalita et al. (2009)/Uttar Pradesh^61^	2004–2006	HBSR	198	198	NA	NA	6
Nagaraja et al. (2009)/Karnataka^62^	2005	HBSR	1174	797	148	NA	5
Sridharan et al. (2009)/Kerala^63^	2005	PBSR	541	311	61	169	7
Dalal et al. (2008)/Maharashtra^64^	2005–2006	PBSR	456	366	81	9	4
Lipska et al. (2007)/Kerala^65^	2002	HBSR	214	97	NM	117	8
Dalal (2006)/Across India^66^	2002–2004	HBSR	2162	1656	461	45	5
Bhattacharya et al. (2005)/West Bengal^67^	1992–1998	PBSR	128	NM	NM	NM	8
Pandiyan et al. (2005)/Tamil Nadu^68^	2003–2004	HBSR	402	NM	NM	NM	6
Mehndiratta et al. (2004)/New Delhi^69^	1988–1997	HBSR	127	109	18	NA	5
Kaul et al. (2000)/Telengana^70^	2000–2001	HBSR	893	893	NA	NA	5
Nayak et al. (1997)/Kerala^71^	1988–1994	HBSR	177	125	9	43	6
Razdan et al. (1989)/Jammu and Kashmir^72^	1986	PBSR	91	NM	NM	NM	2
Chopra and Prabhakar (1979)/Chandigarh^73^	1970–1977	HBSR	251	109	64	78	3

**Abbreviations:** NA, not applicable; NM, not mentioned; PBSR, population-based stroke registry; HBSR, hospital-based stroke register; JBI Score, JBI critical appraisal checklist for analytical cross-sectional studies (score range: 0–8).

### Description of Studies Included the Meta-Analysis

The characteristics of the studies included in the meta-analysis are summarized in Table 2. The meta-analysis included 36 studies, out of which two studies^31, 66^ were multicentric and the remaining included data from various states of the Indian subcontinent (North: 13,^13, 17, 20, 29, 33, 39, 49, 51, 54–56, 69, 61^ South: 14,^14, 19, 23, 40, 45, 48, 53, 59, 62, 63, 65, 68, 70, 71^ East: 3,^28, 36, 6^ West: 2,^47, 57^ and Central: 2^25, 41^). The majority of the studies were published in the past decade.^13, 14, 17, 19, 20, 23, 25, 28, 29, 31, 36, 39–41, 45, 47–49, 51, 53–57, 59^ Overall, 18,315 stroke patients were included, in which the predominant proportion of subjects were males (64%) compared to females (36%). The mean age of the participants was 53.84 ± 9.3 years.

**Table 2.** Lifestyle-Related Risk Factors of Stroke Based on the Included Studies in the Meta-Analysis (*N* = 36).

Author(Year of Publication)/Study Region	Mean Age ± SD(Years)	Male/Female	Sample Size^#^	HTN	DM	Tobacco Use	Alcohol Use	Dyslipidemia	IHD/CAD	RHD/VHD	AF	Others
Singla et al. (2022)/Punjab^13^	NM	1811/1137 (2948)	1736	1506	777	417	887	347	203	58	184	Postpartum-7
Jayadevappa and Ravishankar (2021)/Karnataka^14^	59.6 ± 6.1	140/60	200	32	12	44	80	132	NM	NM	NM	D & H-120, T & A-80
Kumar et al. (2021)/Uttarakhand^17^	55.25 ± 1.32	44/4	48	33	NM	24	15	NM	NM	NM	NM	Hyperhomocysteinemia-12
Prabhakar et al. (2020)/Telengana^19^	61.63	100/44	144	92	24	53	83	NM	NM	NM	NM	Obesity-50
Kaur et al. (2020)/Rajastan^20^	60.46 ± 14.84	217/143	360	189	68	73	NM	93	27	–	–	Anemia-29
Karri and Ramasamy (2019)/Tamil Nadu^23^	38.9 ± 5.74	137/49	186	69	55	92	88	105	29	NM	NM	Hyperhomocysteinemia-23
Panwar et al. (2019)/Madhya Pradesh^25^	31.70 ± 7.42	29/21	50	16	12	20	16	NM	1	NM	NM	Overweight-8 Homocystinaemia-6
Behera and Mohanty (2019)/Odisha^28^	61.4 ± 13.1	287/194	481	336	57	140	67	249	1	2	3	D & H-46, CKD-105, Anemia-128
Pathak et al. (2018)/New Delhi^29^	50.3	200/60	260	169	60	88	64	NM	NM	NM	NM	Myocardial infarction-38
Sylaja et al. (2018)/Across India^31^	58.3 ± 14.7	1389/699	2066	1257	737	668	707	298	349	115	82	NA
Kaur et al. (2017)/Punjab^33^	59 ± 15	3124/1865 (4989)	3330	2849	1564	573	1835	631	344	53	352	Postpartum-13
Mahanta et al. (2018)/Assam^36^	54.3 ± 13	273/177	450	163	52	319	242	NM	3	1	4	NM
Kumar and Rai (2017)/Uttar Pradesh^39^	NM	59/41	100	51	12	36	4	NM	4	3	4	NM
Manorenj et al. (2016)/Telengana^40^	54	69/31	100	83	34	43	61	59	13	NM	NM	Obesity-21, Physical inactivity-28
Nayak et al. (2016)/Madhya Pradesh^41^	NM	73/31	104	70	29	8	11	NM	5	NM	NM	NM
Subha et al. (2015)/Kerala^45^	65.30 ± 12.80	55/45	100	83	49	41	21	24	48	10	10	Physical inactivity-37
Kawle et al. (2015)/Maharashtra^47^	NM	73/31	104	70	29	8	11	NM	5	NM	NM	NM
Shravani et al. (2015)/Karnataka^48^	50	73/27	100	70	27	24	20	22	15	NM	NM	NM
Renjen et al. (2015)/New Delhi^49^	57.1 ± 1.7	165/79	244	139	85	95	NM	58	44	NM	13	NM
Gupta et al. (2014)/Chandigarh^51^	59.9 ± 11.2	49/24	73	64	42	21	NM	63	17	NM	4	Obesity-40
Sorganvi et al. (2014)/Karnataka^53^	62.8	59/41	100	62	38	49	32	66	NM	NM	NM	Obesity-84
Dash et al. (2014)/New Delhi^54^	38.9 ± 7.1	NM	440	196	61	42	42	115	24	56	29	Cardiomyopathy-12
Kulshrestha and Vidyanand (2013)/Uttar Pradesh^55^	NM	92/65	157	26	14	6	NM	9	NM	1	1	D & H-23, Anemia-54
Singh et al. (2013)/Punjab^56^	57.3 ± 13.8	650/506	1156	734	454	246	832	220	NM	NM	96	NM
Deoke et al. (2012)/Maharashtra^57^	59.30 ± 12.44	65/36	101	47	23	61	20	NM	14	NM	NM	Physical inactivity-25, Overweight-16
Kumar et al. (2011)/Karnataka^59^	NM	74/35	109	79	59	76	53	56	NM	NM	NM	Obesity-53, Homocystienemia-7,
Kalita et al. (2009)/Uttar Pradesh^61^	53.5 ± 15.9	162/36	198	109	49	61	57	23	NM	NM	NM	Overweight-57
Nagaraja et al. (2009)/Karnataka^62^	54.5 ± 17.0	787/387	1174	563	271	383	295	NM	NM	NM	114	D & H-217
Sridharan et al. (2009)/Kerala^63^	67 (Median)	262/279	541	450	271	70	NM	138	NM	NM	42	NM
Lipska et al. (2007)/Kerala^65^	NM	141/73	214	102	47	79	NM	66	NM	NM	NM	NM
Dalal (2006)/Across India^66^	NM	1576/586	2162	611	88	NM	NM	NM	82	NM	NM	D & H-391, D & H and CAD-154
Bhattacharya et al. (2005)/West Bengal^67^	61	60/68	128	40	18	72	NM	NM	17	NM	NM	NM
Pandiyan et al. (2005)/Tamil Nadu^68^	61.7 ± 13.4	265/137	402	289	200	95	NM	105	136	14	13	Anemia-40
Mehndiratta et al. (2004)/New Delhi^69^	31.97	61/66	127	25	8	29	5	36	9	16	NM	Homocystenemia-1
Kaul et al. (2000)/Telengana^70^	56.9	NM	893	553	250	25	NM	NM	NM	NM	NM	NM
Nayak et al. (1997)/Kerala^71^	34.7 ± 8	135/42	177	32	13	64	30	18	NM	NM	NM	NM

**Note:**^#^ Calculated based on the analysis of risk factors.

**Abbreviations:** PHo/S, previous history of stroke; FHo/S, family history of stroke; NM, not mentioned; HTN, hypertension; DM, diabetic mellitus; CAD/IHD, coronary artery disease/ischemic heart disease; CKD, chronic kidney disease; AF, atrial fibrillation; RHD/VHD, rheumatic heart disease/valvular heart disease; D & H, diabetes and hypertension; T & A, tobacco and alcohol use.

The total score of risk of bias assessment according to the JBI critical appraisal checklist for analytical cross-sectional studies is eight, and the individual scores of the included studies in the systematic review ranged from two to eight. We took an arbitrary cutoff based on the mean and the median scores as there is no specified cutoff for the classification of studies for risk of bias. The mean score of the 38 studies was 5.06, and the median score was five. We considered five as a cutoff point. Finally, 36 studies were included in the meta-analysis. Most studies had credible information about the eligibility criteria, study population, setting, and outcome measures. The reporting structure of the measurement of risk factors for stroke and the influence of confounding variables was poorly followed in more than half of the studies. The details of the quality assessment of the studies using the JBI checklist are described in Table 3.

**Table 3.** Risk of Bias Assessment of Included Studies.

Author (Year of Publication)	Study Design	Q1	Q2	Q3	Q4	Q5	Q6	Q7	Q8	Total	Meta-Analysis
Singla et al. (2022)	PBSR	1	1	UC	1	1	1	1	1	7	Included
Jayadevappa and Ravishankar (2021)	HBSR	1	1	UC	1	1	0	1	0	5	Included
Kumar et al. (2021)	HBSR	1	1	UC	1	1	0	1	0	5	Included
Prabhakar et al. (2020)	PBSR	1	1	1	1	1	1	1	1	8	Included
Kaur et al. (2020)	HBSR	1	1	UC	1	1	0	1	0	5	Included
Karri and Ramasamy (2019)	HBSR	1	1	UC	1	1	0	1	0	5	Included
Panwar et al. (2019)	HBSR	1	1	UC	1	1	0	1	0	5	Included
Behera and Mohanty (2019)	HBSR	1	1	1	1	0	0	1	0	5	Included
Pathak et al. (2018)	HBSR	1	1	1	1	0	0	1	0	5	Included
Sylaja et al. (2018)	HBSR	1	1	1	1	0	0	1	0	5	Included
Kaur et al. (2017)	PBSR	1	1	1	1	1	1	1	1	8	Included
Mahanta et al. (2018)	HBSR	1	1	UC	1	1	1	1	1	7	Included
Kumar and Rai (2017)	HBSR	1	1	0	1	1	0	1	0	5	Included
Manorenj et al. (2016)	HBSR	UC	1	1	1	1	1	1	1	7	Included
Nayak et al. (2016)	HBSR	1	1	1	1	1	0	1	0	6	Included
Subha et al. (2015)	HBSR	1	1	1	1	1	1	1	1	8	Included
Kawle et al. (2015)	HBSR	1	1	1	1	1	UC	1	0	6	Included
Shravani et al. (2015)	HBSR	1	1	0	1	1	1	1	1	7	Included
Renjen et al. (2015)	HBSR	1	1	0	1	1	1	1	1	7	Included
Gupta et al. (2014)	HBSR	1	1	1	1	0	0	1	0	5	Included
Sorganvi et al. (2014)	HBSR	1	1	1	1	1	1	1	1	8	Included
Dash et al. (2014)	HBSR	1	1	UC	1	1	1	1	0	6	Included
Kulshrestha and Vidyanand (2013)	HBSR	1	1	1	1	0	0	1	0	5	Included
Singh et al. (2013)	HBSR	1	1	1	1	1	1	1	1	8	Included
Deoke et al. (2012)	HBSR	1	1	1	1	1	1	1	1	8	Included
Kumar et al. (2011)	HBSR	1	1	1	1	1	UC	1	UC	6	Included
Kalita et al. (2009)	HBSR	1	1	1	1	1	UC	1	UC	6	Included
Nagaraja et al. (2009)	HBSR	1	1	1	1	0	0	1	0	5	Included
Sridharan et al. (2009)	PBSR	1	1	1	1	1	1	1	UC	7	Included
Lipska et al. (2007)	HBSR	1	1	1	1	1	1	1	1	8	Included
Dalal (2006)	HBSR	1	1	1	1	UC	UC	1	UC	5	Included
Bhattacharya et al. (2005)	PBSR	1	1	1	1	1	1	1	1	8	Included
Pandiyan et al. (2005)	HBSR	1	1	1	1	UC	UC	1	1	6	Included
Mehndiratta et al. (2004)	HBSR	1	1	1	1	UC	UC	1	0	5	Included
Kaul et al. (2000)	HBSR	1	1	UC	1	1	0	1	0	5	Included
Nayak et al. (1997)	HBSR	1	1	1	1	1	UC	1	0	6	Included

**Note:** Mean, 5.06; Median, 5.

**Abbreviations:** PBSR, population-based stroke registry; HBSR, hospital-based stroke register; UC, unclear.

### Lifestyle Risk Factors for Stroke

**Figure 2.**
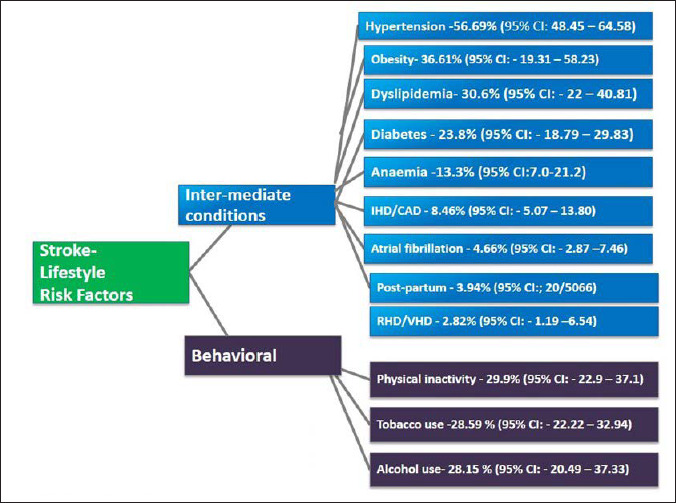
The Distribution of Lifestyle Risk Factors Among Patients with Stroke in India.

Stroke being an end-stage disease, we distributed the risk factors for stroke as behavioral risk factors and intermediate conditions (Figure 2). Hypertension (56.69%; 95% CI [48.45, 64.58]; *n* = 36 studies), obesity (36.61%; 95% CI [19.31, 58.23]; *n* = 8 studies), dyslipidemia (30.6%; 95% CI [22, 40.81]; *n* = 23 studies), and diabetes mellitus (23.8%; 95% CI [18.79, 29.83]; *n* = 35 studies) were identified as the leading intermediate conditions associated with stroke. The presence of various forms of anemia was associated with 13.3% (95% CI [7.0, 21.2]; *n* = 9 studies) of the patients with stroke. The occurrence of ischemic heart disease or coronary artery diseases was reported in 8.46% (95% CI [5.07, 13.80]; *n* = 22 studies) of the study subjects, and 2.82% (95% CI [1.19, 6.54]; *n* = 11 studies) of the participants had rheumatic heart disease or valvular heart disease. An ECG diagnosis of atrial fibrillation was identified for 4.66% (95% CI [2.87, 7.46]; *n* = 15 studies) of patients. Approximately 4% (3.94%; 95% CI [2.0, 6.0]) of the patients experienced an episode of stroke during postpartum period.

The physical inactivity–29.9% (95% CI [22.9, 37.1]), history of tobacco use, both smoking and smokeless forms (28.59%; 95% CI [22.22, 32.94]), and alcohol use (28.15%; 95% CI [20.49, 37.33]) were the behavioral risk factors for stroke in this setting.

We used the DerSimonian and Laird method of random-effects models to calculate the pooled estimates as there was a significant heterogeneity in the outcome measures (hypertension–*I*^2^ = 98.8%, tau squared = 0.92, Q = 2932.05, *P* < .001; diabetes–*I*^2^ = 97.9%, tau squared = 0.74, Q = 1641.78, *P* < .001; dyslipidemia–*I*^2^ = 97.6%, tau squared = 1.03, Q = 909.22, *P* < .001; atrial fibrillation–*I*^2^ = 90.9%, tau squared = 0.70, Q = 154.50, *P* < .001; tobacco–*I*^2^ = 97.3%, tau squared = 0.09, Q = 1238.27, *P* < .001; alcohol–*I*^2^ = 98.2%, tau squared = 1.02, Q = 1422.63, *P* < .001; IHD/CAD–*I*^2^ = 96%, tau squared = 1.40, Q = 525.33, *P* < .001; RHD/VHD–*I*^2^ = 94.5%, tau squared = 1.50, Q = 182.64, *P* < .001; obesity–*I*^2^ = 94.3%, tau squared = 1.04, Q = 122.53, *P* < .001; previous history of stroke–*I*^2^ = 96%, tau squared = 0.61, Q = 447.43, *P* < .001; family history of stroke–*I*^2^ = 92.6%, tau squared = 0.70, Q = 162.83, *P* < .001). The pooled analysis of the proportion of individual risk factors and heterogeneity are depicted in supplementary materials (Figure S1.1–S1.9)

With the exception of the proportion of studies that estimated diabetes (*P* = .01), dyslipidemia (*P* = .02), alcohol (*P* = .01), and atrial fibrillation (*P* = .01), Egger’s test revealed no publication bias in the outcome measures (hypertension: *P* = .53; tobacco: *P* = .39; RHD/VHD: *P* = .52; IHD/CAD: *P* = .39; previous history of stroke *P* = .12; family history of stroke *P* = .44). The funnel plot regarding the publication bias of individual risk factors based on the included studies is presented in supplementary materials (Figure S2.1–S2.9).

## Discussion

Reducing the burden of stroke in the Indian population requires the identification of modifiable risk factors, and the current meta-analysis provides an aggregate of the distribution of various lifestyle-related risk factors for patients with stroke in the Indian setting. We identified that hypertension (56.69%), obesity (36.61%), dyslipidemia (30.6%), and diabetes mellitus (23.8%) are the leading intermediate conditions associated with stroke. However, physical inactivity (29.9%), history of tobacco use (28.59%), and alcohol use (28.15%) were reported as the behavioral risk factors for stroke in this setting. Referring to some previous studies, there are conflicting results regarding pooled estimates of the risk factors for stroke across Asian countries.^74^ Hypertension remains the most common vascular risk factor for stroke in the Asian population^74–76^ which is consistent with current study findings.

However, the comparisons of the aggregate estimate of the lifestyle risk factors of stroke in this setting should be interpreted based on several contextual factors. First, the high or low frequencies of the occurrence of the risk factors for the noncommunicable disease of a country need to be considered while estimating the specific risk factors for stroke in other countries. For example, a high prevalence of hypertension is seen in Mongolia and Pakistan, which is low in Korea and Singapore.^74^ Therefore, a countrywide comparison would be made based on the magnitude of the risk factors predisposing stroke. Second, most of the information on the risk factors among stroke patients was derived from HBSR studies in which data were collected at differing time points with varying definitions limiting its generalization for the estimation of risk factors across countries.^77, 78^ It is worth noting that estimating stroke risk based on valid risk scoring systems is of pivotal importance for better understanding risk factors to maximize the efficacy of risk reduction efforts.^79^

Currently, the burden of stroke is increasing in India,^80^ and the findings of this meta-analysis reflect a comprehensive report on the trends of risk factors for stroke in India over a long period. Although several HBSR and PBSR studies were conducted in different parts of India, there is a dearth of evidence of a systematic summary of the risk factor profile of stroke in this setting. This study provides robust estimates of the lifestyle risk factor of stroke in India from 1994 to 2019. The mean age of the participants was 53.84 ± 9.3 years, and a predominant proportion of subjects were males (64%). In contrast to our findings, earlier epidemiological studies in India have found hypertension, diabetes, and cigarette smoking as the leading lifestyle risk factors for stroke.^81^ One of the reasons for this change in the risk factor profile for stroke might be because of the varied epidemiological transition among the different states of India.^82^ The current findings emphasize that, although the proportion of risk factors for stroke varied considerably across the states of India, the prevalence of hypertension remains the pivotal risk factor across all state groups since 1994. The burden of stroke in the developing world is likely to increase substantially, partly because of ongoing demographic changes, including the aging of the population and health transitions in these countries.^83^

The current meta-analysis indicates an urgent need for controlling the vascular and lifestyle risk factors of stroke by focusing more on the public campaign to build the protective factors against cerebrovascular diseases in this setting. There was a significant inconsistency among the included studies as the level of heterogeneity was high (*I*^2^ > 94%). The risk of bias assessment of the included studies has implications for the generalization of our findings. Therefore, we exclusively selected stroke epidemiological studies with a low risk of bias conducted in the Indian setting. The current findings provide an evidence base to successfully meet the challenges while devising appropriate strategies to curtail the strategies targeted for risk factor modification.

## Strength and Limitations

The primary uniqueness of this study is the novelty of a meta-analysis reflecting the pooled estimate of the proportion of various risk factors of stroke from an Indian perspective. There are certain limitations to generalizing our findings. The results are purely based on observational studies with methodological limitations, such as sampling bias and respondent bias. The level of heterogeneity of the included studies was high because of differences in the study contexts. There might be a chance of contamination of the study subjects, as our estimation is based on the pooled analysis of both hospital and population-based studies, including stroke register studies. The data on clustering risk factors for stroke were not estimated as it was poorly reported in most studies. Despite the limitations, the current meta-analysis provides robust estimates of the lifestyle-related risk factor of stroke in India based on the observational studies conducted from 1994 to 2019.

## Conclusion

The present meta-analysis elucidates the overall estimates of lifestyle risk factors for patients with stroke in India. Estimating the pooled analysis of stroke risk factors is crucial to predict the imposed burden of the illness and ascertain the treatment and prevention strategies for controlling the modifiable risk factors in this setting.

## Supplemental Material

Supplemental material for this article is available online. Click here for additional data file. Supplemental Material for The Distribution of Lifestyle Risk Factors Among Patients with Stroke in the Indian Setting: Systematic Review and Meta-Analysis by Biji P. Varkey, Jaison Joseph, Abin Varghese, Suresh K. Sharma, Elezebeth Mathews, Manju Dhandapani, Venkata Lakshmi Narasimha, Radha Kuttan, Saleena Shah, Surekha Dabla and Sivashanmugam Dhandapani, in Annals of Neurosciences
