# Supplementary material for: The Distribution of Lifestyle Risk Factors Among Patients with Stroke in the Indian Setting: Systematic Review and Meta-Analysis
Source: Ann Neurosci. 2022 Aug 8;30(1):40–53. doi: 10.1177/09727531221115899 (PMC10259149; doi:10.1177/09727531221115899)
Supplement: Supplemental material for this article is available online. [file sj-pdf-1-aon-10.1177_09727531221115899.pdf]

Supplementary Material (1) -Search Strategy using keywords

Example of search terms used in PubMed

**Search concept**	**MeSH terms and keywords**
Stroke	“stroke” [MeSH] OR "ischemic stroke"[MeSH] OR "hemorrhagic stroke"[MeSH] OR "thrombotic stroke" [MeSH] "embolic stroke"[MeSH] OR “acute stroke” [tiab] OR "ischemic stroke" [tiab] OR "hemorrhagic stroke" [tiab] OR "thrombotic stroke" [tiab] OR "embolic stroke" OR “cerebrovascular accident” [tiab] “cerebral stroke” [tiab]
Risk factors	"prevalence" [MeSH] OR "epidemiology" [MeSH] OR “risk factors” [MeSH] OR "cardiometabolic risk factors" [MeSH] OR "hypertension" [MeSH] OR "diabetes mellitus" [MeSH] OR "alcoholism" [MeSH] OR "alcohol drinking" [MeSH] OR "dyslipidemias" [MeSH] OR "smoking" [MeSH] "tobacco use" [MeSH] OR “risk factors” [tiab] OR “health correlates” [tiab] OR "cardiometabolic risk factors"[tiab] OR "hypertension" [tiab] OR "diabetes mellitus" [tiab] OR “alcohol use disorder [tiab] OR “alcohol abuse” [tiab] OR “alcohol dependent” [tiab] OR "dyslipidemias" [tiab] OR "smoking"[tiab] OR "tobacco use"[tiab] “tobacco consumption” [tiab]
Patients	"patients"[MeSH] OR “patient” [tiab] OR “clients” [tiab]
India	"india"[MeSH] OR “India” [tiab] OR “South-east Asia” [tiab] OR “low- and middle-income countries” [tiab]

Supplementary Material (S 1.1 - 1.11 Figure)

The pooled analysis of the proportion of individual risk factors and heterogenity

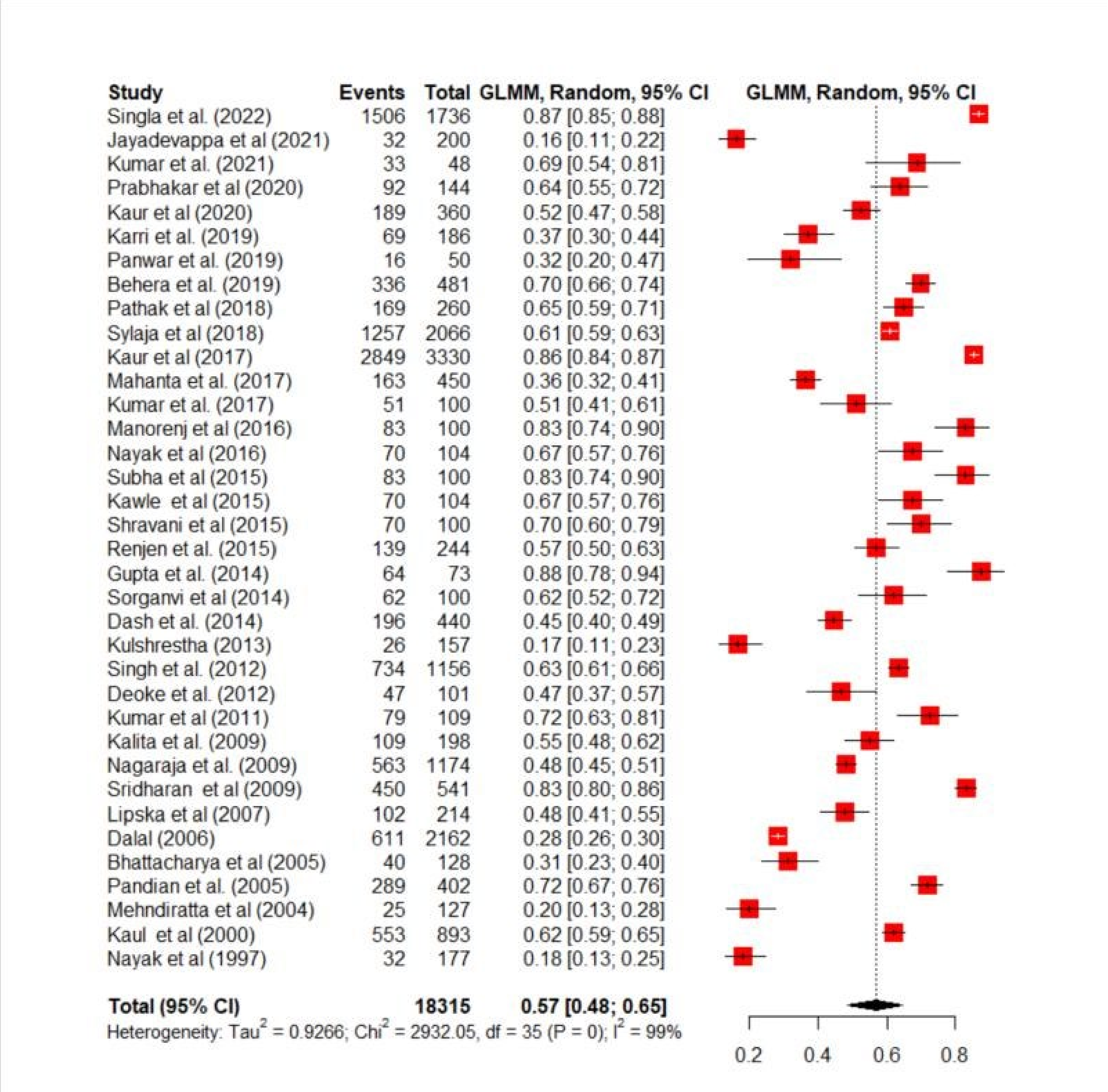

S 1.1 Figure Pooled analysis of the proportion of hypertension among patients with stroke

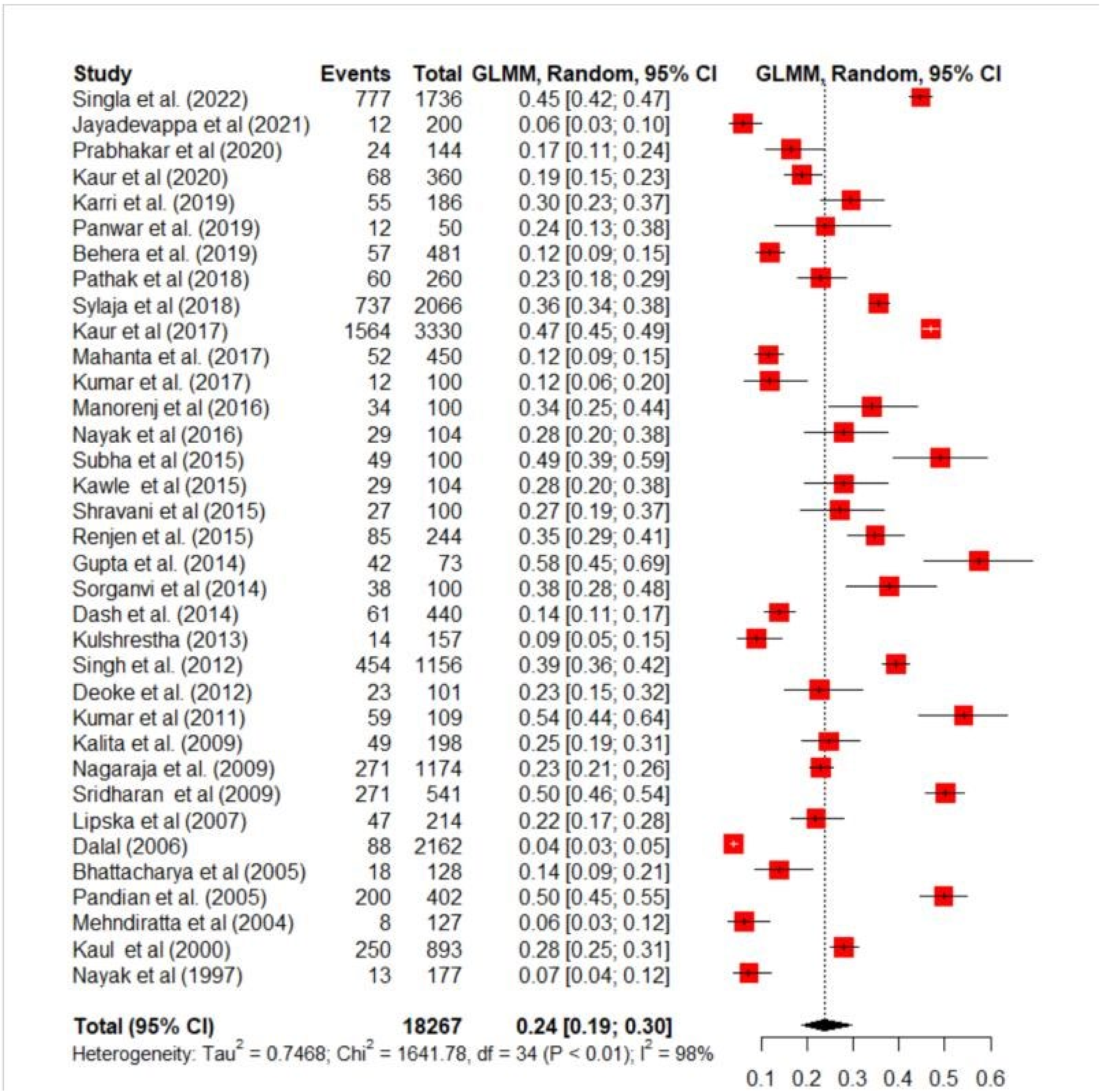

S1.2 Figure Pooled analysis of the proportion of diabetes among patients with stroke

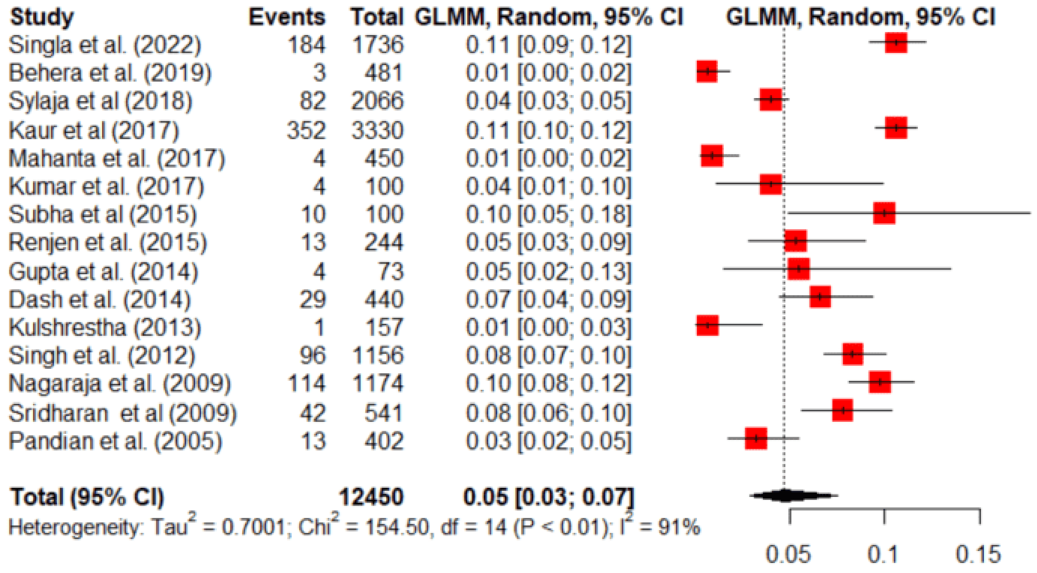

S1.3 Figure Pooled analysis of the proportion of atrial fibrillation among patients with stroke

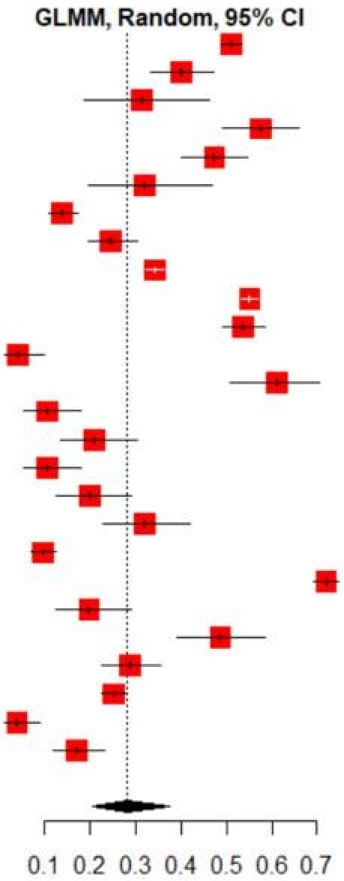

Study	Events	Total	GLMM, Random, 95% CI
Singla et al. (2022)	887	1736	0.51 [0.49; 0.53]
Jayadevappa et al (2021)	80	200	0.40 [0.33; 0.47]
Kumar et al. (2021)	15	48	0.31 [0.19; 0.46]
Prabhakar et al (2020)	83	144	0.58 [0.49; 0.66]
Karri et al. (2019)	88	186	0.47 [0.40; 0.55]
Panwar et al. (2019)	16	50	0.32 [0.20; 0.47]
Behera et al. (2019)	67	481	0.14 [0.11; 0.17]
Pathak et al (2018)	64	260	0.25 [0.20; 0.30]
Sylaja et al (2018)	707	2066	0.34 [0.32; 0.36]
Kaur et al (2017)	1835	3330	0.55 [0.53; 0.57]
Mahanta et al. (2017)	242	450	0.54 [0.49; 0.58]
Kumar et al. (2017)	4	100	0.04 [0.01; 0.10]
Manorenj et al (2016)	61	100	0.61 [0.51; 0.71]
Nayak et al (2016)	11	104	0.11 [0.05; 0.18]
Subha et al (2015)	21	100	0.21 [0.13; 0.30]
Kawle et al (2015)	11	104	0.11 [0.05; 0.18]
Shravani et al (2015)	20	100	0.20 [0.13; 0.29]
Sorganvi et al (2014)	32	100	0.32 [0.23; 0.42]
Dash et al. (2014)	42	440	0.10 [0.07; 0.13]
Singh et al. (2012)	832	1156	0.72 [0.69; 0.75]
Deoke et al. (2012)	20	101	0.20 [0.13; 0.29]
Kumar et al (2011)	53	109	0.49 [0.39; 0.58]
Kalita et al. (2009)	57	198	0.29 [0.23; 0.36]
Nagaraja et al. (2009)	295	1174	0.25 [0.23; 0.28]
Mehndiratta et al (2004)	5	127	0.04 [0.01; 0.09]
Nayak et al (1997)	30	177	0.17 [0.12; 0.23]
**Total (95% CI)**		**13141**	**0.28 [0.20; 0.37]**

Heterogeneity: $Tau^2 = 1.0255$; $Chi^2 = 1422.63$, df = 25 ($P < 0.01$); $I^2 = 98\%$

S1.4 Figure Pooled analysis of the proportion of alcohol use among patients with stroke

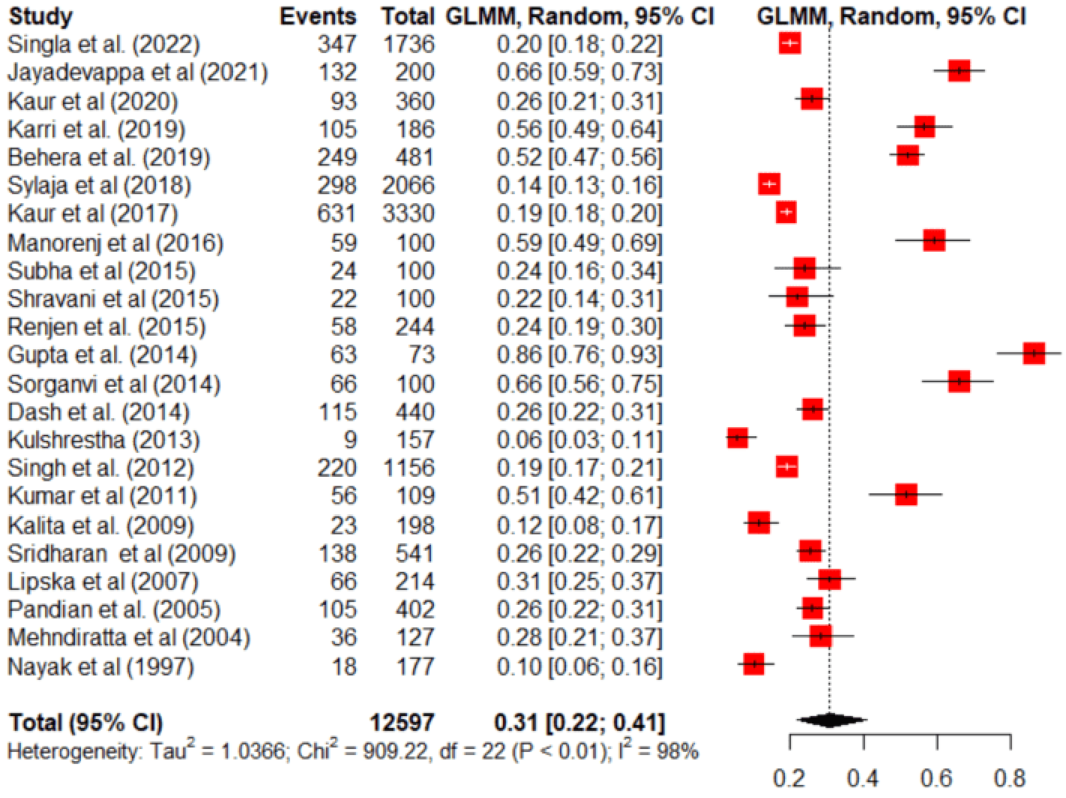

S1.5 Figure Pooled analysis of the proportion of dyslipidemia among patients with stroke

Study	Events	Total	GLMM, Random, 95% CI
Singla et al. (2022)	203	1736	0.12 [0.10; 0.13]
Kaur et al (2020)	27	360	0.07 [0.05; 0.11]
Karri et al. (2019)	29	186	0.16 [0.11; 0.22]
Panwar et al. (2019)	1	50	0.02 [0.00; 0.11]
Behera et al. (2019)	1	481	0.00 [0.00; 0.01]
Sylaja et al (2018)	349	2066	0.17 [0.15; 0.19]
Kaur et al (2017)	344	3330	0.10 [0.09; 0.11]
Mahanta et al. (2017)	3	450	0.01 [0.00; 0.02]
Kumar et al. (2017)	4	100	0.04 [0.01; 0.10]
Manorenj et al (2016)	13	100	0.13 [0.07; 0.21]
Nayak et al (2016)	5	104	0.05 [0.02; 0.11]
Subha et al (2015)	48	100	0.48 [0.38; 0.58]
Kawle et al (2015)	5	104	0.05 [0.02; 0.11]
Shravani et al (2015)	15	100	0.15 [0.09; 0.24]
Renjen et al. (2015)	44	244	0.18 [0.13; 0.23]
Gupta et al. (2014)	17	73	0.23 [0.14; 0.35]
Dash et al. (2014)	24	440	0.05 [0.04; 0.08]
Deoke et al. (2012)	14	101	0.14 [0.08; 0.22]
Dalal (2006)	82	2162	0.04 [0.03; 0.05]
Bhattacharya et al (2005)	17	128	0.13 [0.08; 0.20]
Pandian et al. (2005)	136	402	0.34 [0.29; 0.39]
Mehndiratta et al (2004)	9	127	0.07 [0.03; 0.13]
**Total (95% CI)**		**12944**	**0.08 [0.05; 0.14]**

Heterogeneity: $Tau^2 = 1.4070$; $Chi^2 = 525.33$, df = 21 ($P < 0.01$); $I^2 = 96\%$

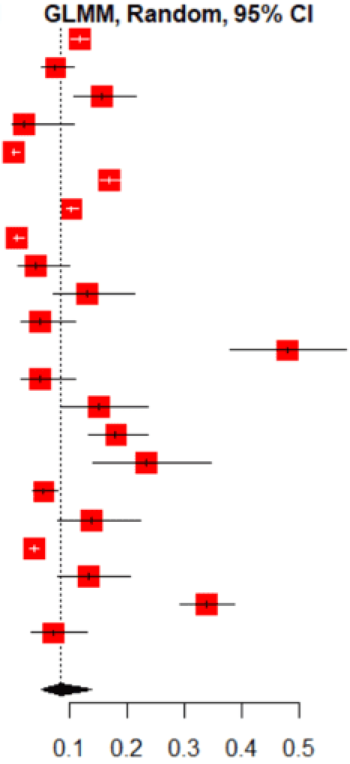

S1.6 Figure Pooled analysis of the proportion of ischemic heart disease among patients with stroke

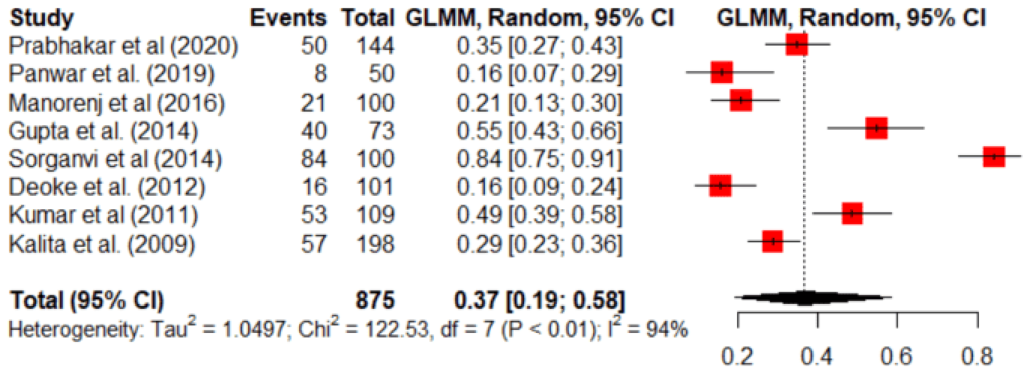

S1.7 Figure Pooled analysis of the proportion of obseity among patients with stroke

Study	Events	Total	GLMM, Random, 95% CI
Singla et al. (2022)	58	1736	0.03 [0.03; 0.04]
Behera et al. (2019)	2	481	0.00 [0.00; 0.01]
Sylaja et al (2018)	115	2066	0.06 [0.05; 0.07]
Kaur et al (2017)	53	3330	0.02 [0.01; 0.02]
Mahanta et al. (2017)	1	450	0.00 [0.00; 0.01]
Kumar et al. (2017)	3	100	0.03 [0.01; 0.09]
Subha et al (2015)	10	100	0.10 [0.05; 0.18]
Dash et al. (2014)	56	440	0.13 [0.10; 0.16]
Kulshrestha (2013)	1	157	0.01 [0.00; 0.03]
Pandian et al. (2005)	14	402	0.03 [0.02; 0.06]
Mehndiratta et al (2004)	16	127	0.13 [0.07; 0.20]
**Total (95% CI)**		**9389**	**0.03 [0.01; 0.07]**

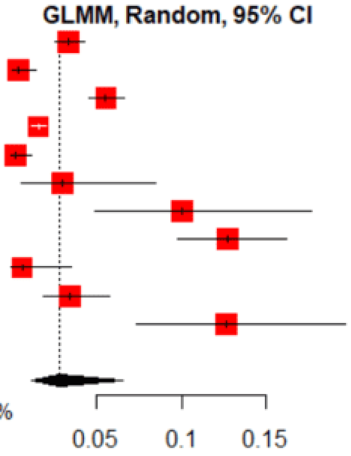

Heterogeneity: $Tau^2 = 1.5006$; $Chi^2 = 182.64$, df = 10 ($P < 0.01$); $I^2 = 95\%$

S1.8 Figure Pooled analysis of the proportion of rheumatic/valvular heart disease among patients with stroke

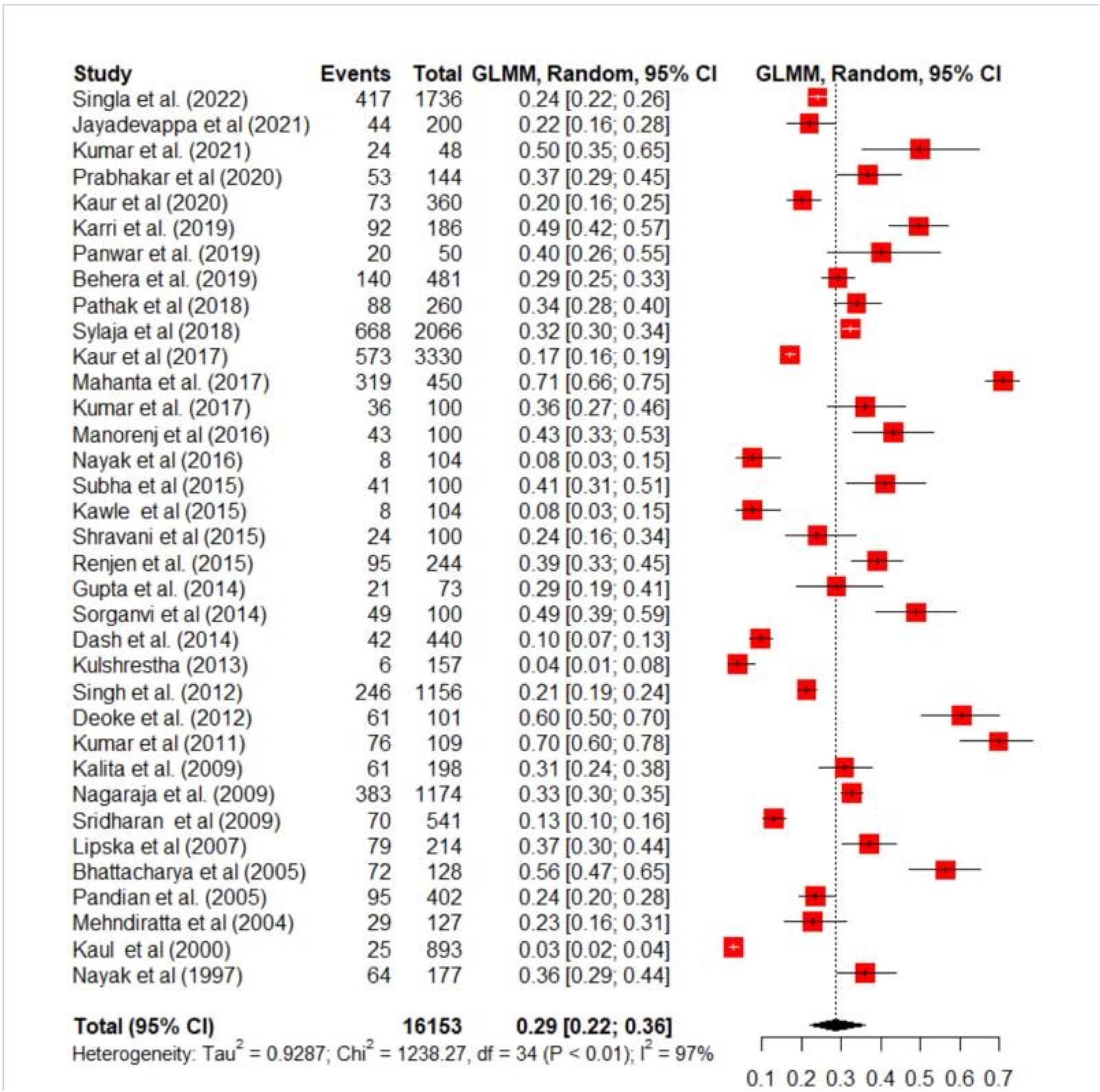

Study	Events	Total	GLMM, Random, 95% CI
Singla et al. (2022)	417	1736	0.24 [0.22; 0.26]
Jayadevappa et al (2021)	44	200	0.22 [0.16; 0.28]
Kumar et al. (2021)	24	48	0.50 [0.35; 0.65]
Prabhakar et al (2020)	53	144	0.37 [0.29; 0.45]
Kaur et al (2020)	73	360	0.20 [0.16; 0.25]
Karri et al. (2019)	92	186	0.49 [0.42; 0.57]
Panwar et al. (2019)	20	50	0.40 [0.26; 0.55]
Behera et al. (2019)	140	481	0.29 [0.25; 0.33]
Pathak et al (2018)	88	260	0.34 [0.28; 0.40]
Sylaja et al (2018)	668	2066	0.32 [0.30; 0.34]
Kaur et al (2017)	573	3330	0.17 [0.16; 0.19]
Mahanta et al. (2017)	319	450	0.71 [0.66; 0.75]
Kumar et al. (2017)	36	100	0.36 [0.27; 0.46]
Manorenj et al (2016)	43	100	0.43 [0.33; 0.53]
Nayak et al (2016)	8	104	0.08 [0.03; 0.15]
Subha et al (2015)	41	100	0.41 [0.31; 0.51]
Kawle et al (2015)	8	104	0.08 [0.03; 0.15]
Shravani et al (2015)	24	100	0.24 [0.16; 0.34]
Renjen et al. (2015)	95	244	0.39 [0.33; 0.45]
Gupta et al. (2014)	21	73	0.29 [0.19; 0.41]
Sorganvi et al (2014)	49	100	0.49 [0.39; 0.59]
Dash et al. (2014)	42	440	0.10 [0.07; 0.13]
Kulshrestha (2013)	6	157	0.04 [0.01; 0.08]
Singh et al. (2012)	246	1156	0.21 [0.19; 0.24]
Deoke et al. (2012)	61	101	0.60 [0.50; 0.70]
Kumar et al (2011)	76	109	0.70 [0.60; 0.78]
Kalita et al. (2009)	61	198	0.31 [0.24; 0.38]
Nagaraja et al. (2009)	383	1174	0.33 [0.30; 0.35]
Sridharan et al (2009)	70	541	0.13 [0.10; 0.16]
Lipska et al (2007)	79	214	0.37 [0.30; 0.44]
Bhattacharya et al (2005)	72	128	0.56 [0.47; 0.65]
Pandian et al. (2005)	95	402	0.24 [0.20; 0.28]
Mehndiratta et al (2004)	29	127	0.23 [0.16; 0.31]
Kaul et al (2000)	25	893	0.03 [0.02; 0.04]
Nayak et al (1997)	64	177	0.36 [0.29; 0.44]
**Total (95% CI)**		**16153**	**0.29 [0.22; 0.36]**

Heterogeneity: $Tau^2 = 0.9287$; $Chi^2 = 1238.27$, df = 34 ($P < 0.01$); $I^2 = 97\%$

S1.9 Figure Pooled analysis of the proportion of tobacco use among patients with stroke

Supplementary Material (S 2.1 - 2.11 Figure)

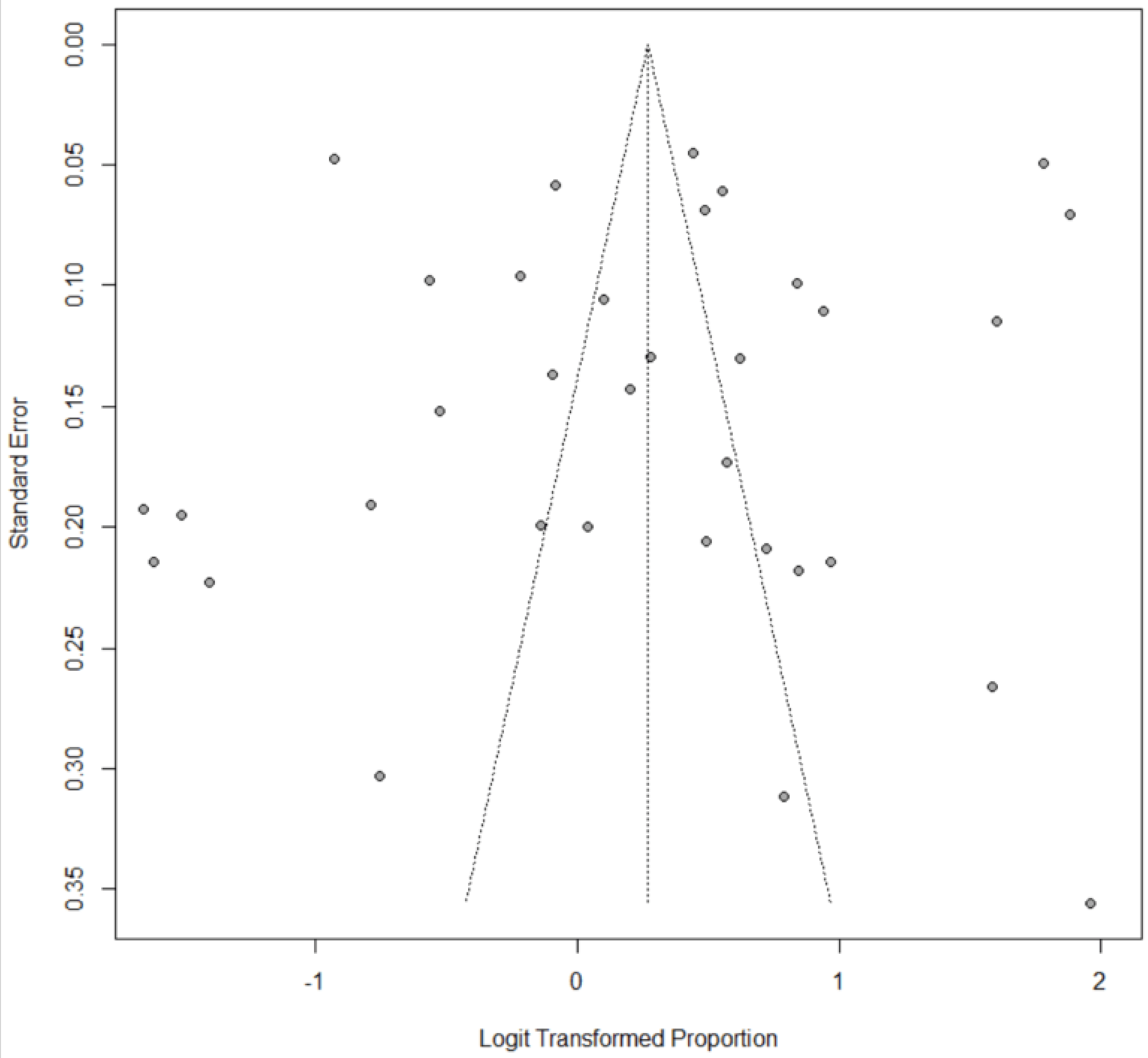

S 2.1 Figure Funnel plot regarding the proportion of hypertension among patients with stroke

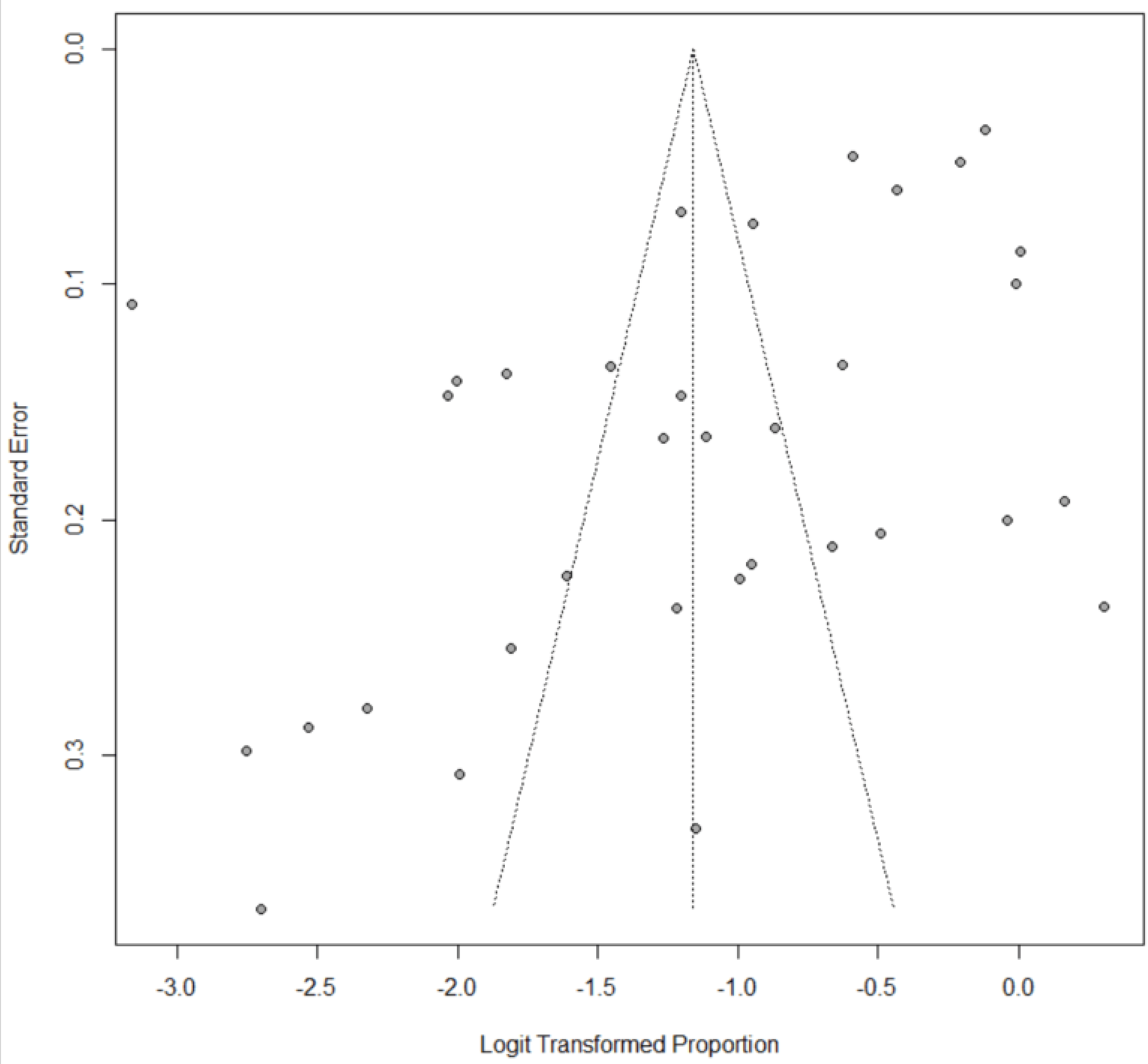

S 2.2 Figure Funnel plot regarding the proportion of diabetes among patients with stroke

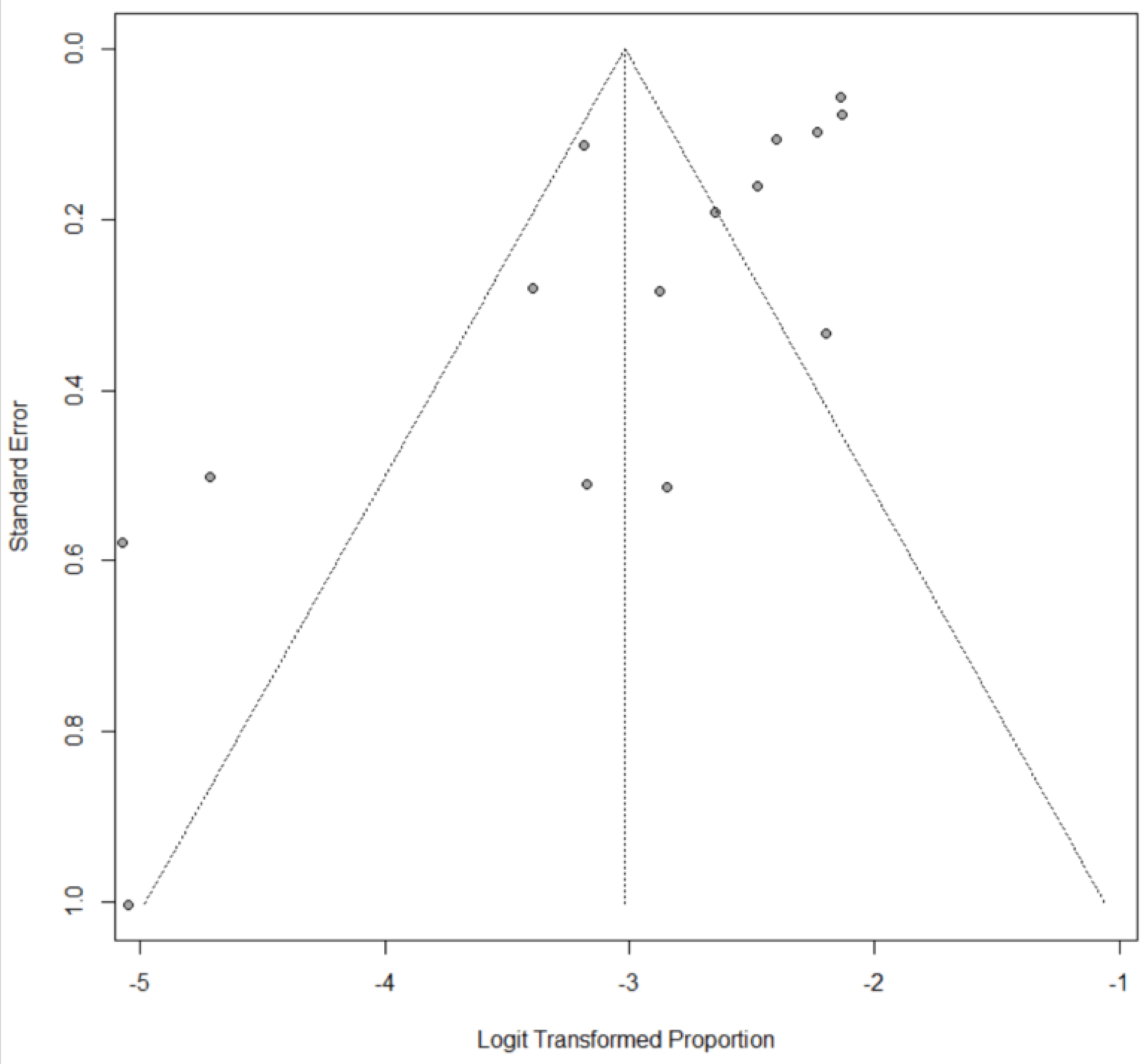

S 2.3 Figure Funnel plot regarding the proportion of atrial fibrillation among patients with stroke

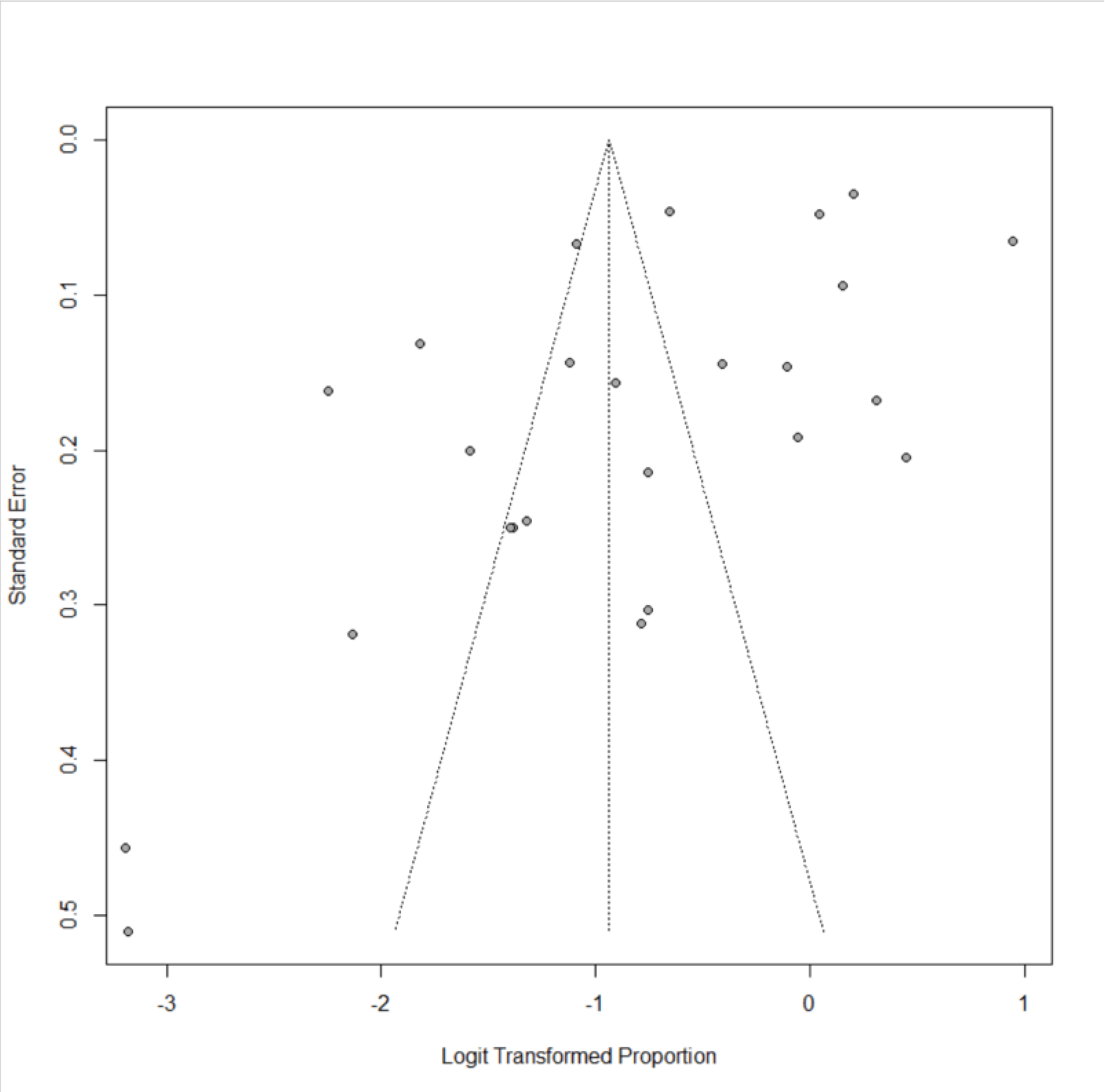

S 2.4 Figure Funnel plot regarding the proportion of alcohol use among patients with stroke

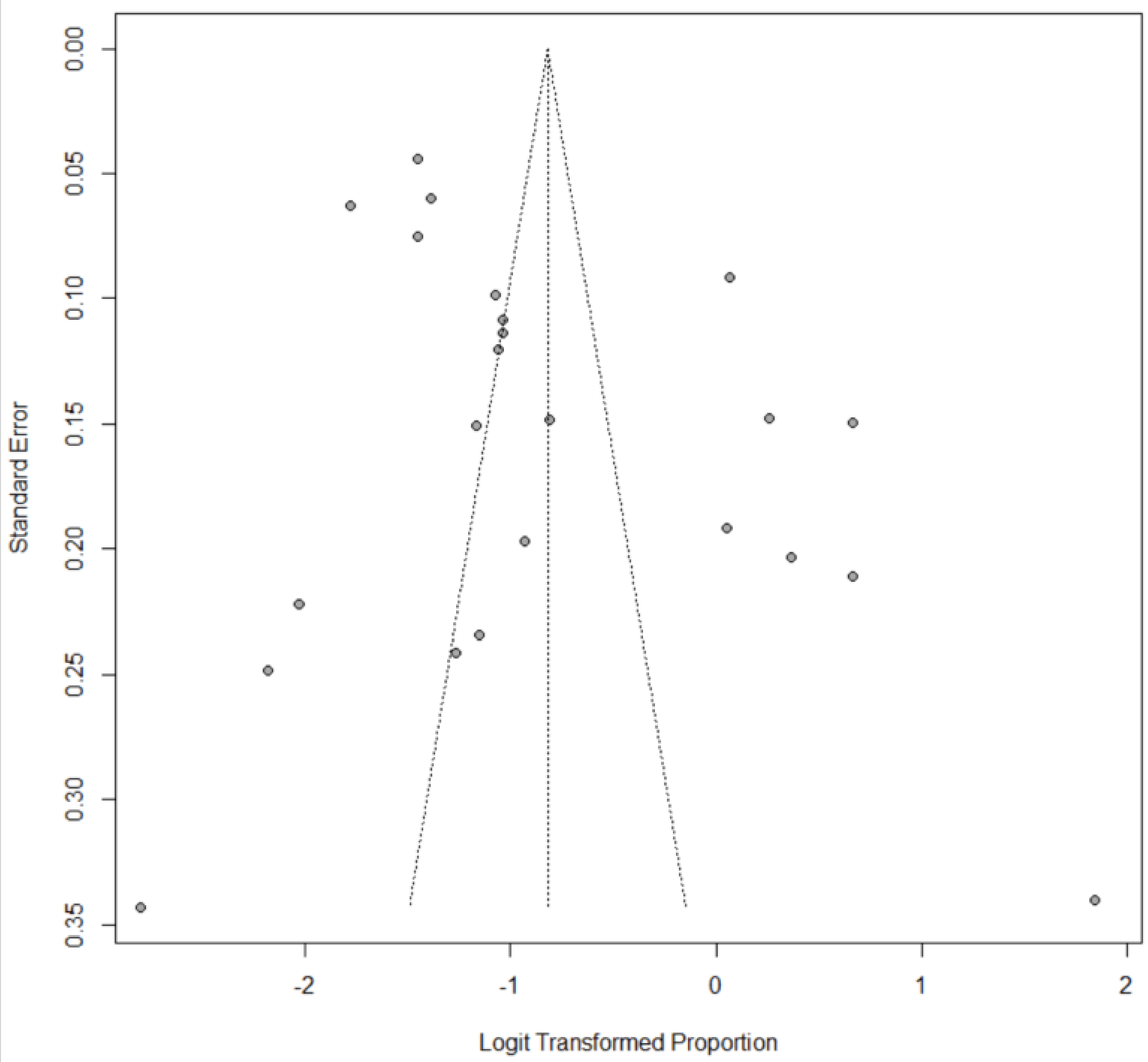

S 2.5 Figure Funnel plot regarding the proportion of dyslipdemia among patients with stroke

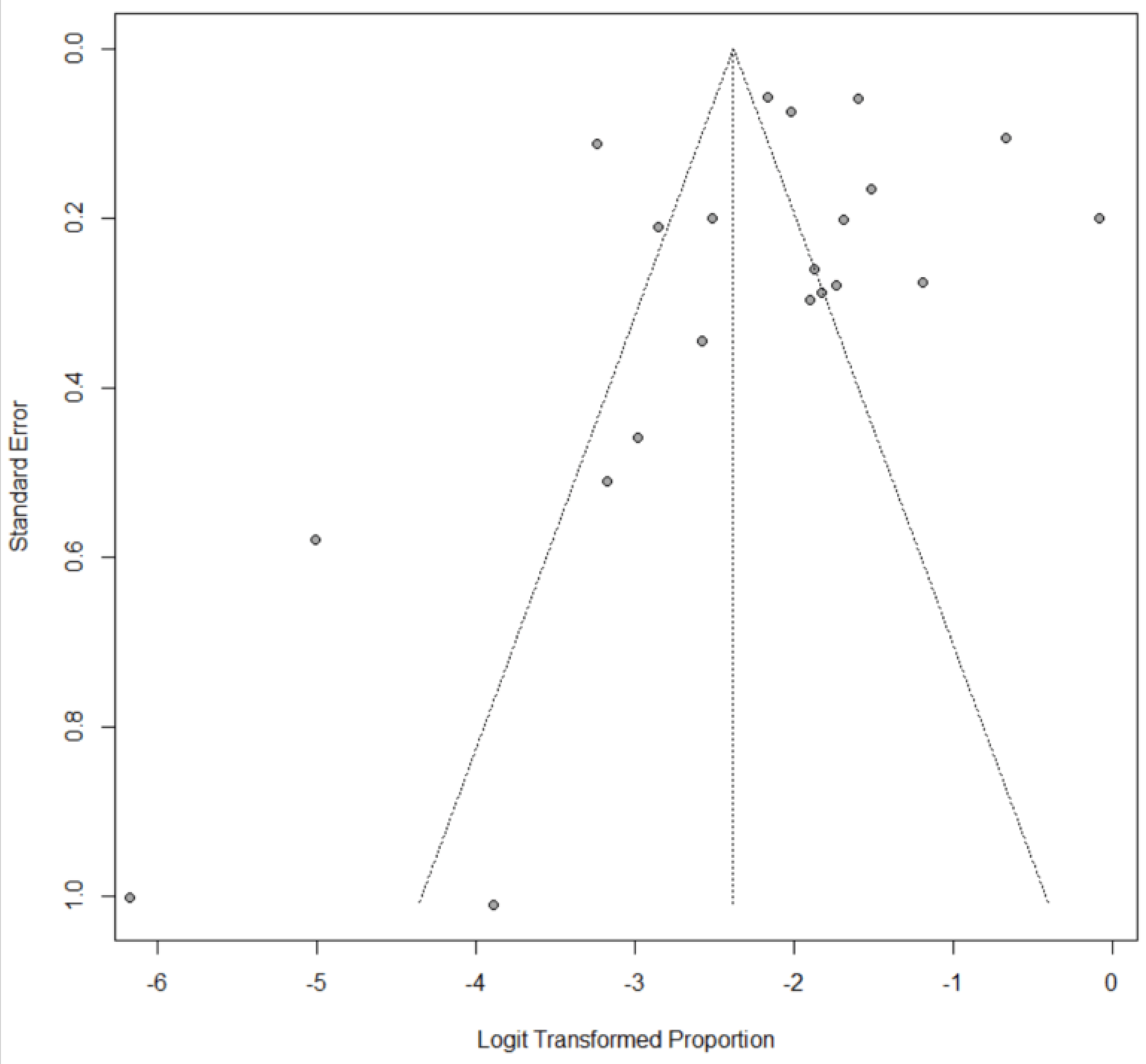

S 2.6 Figure Funnel plot regarding the proportion of ischemic heart diseases among patients with stroke

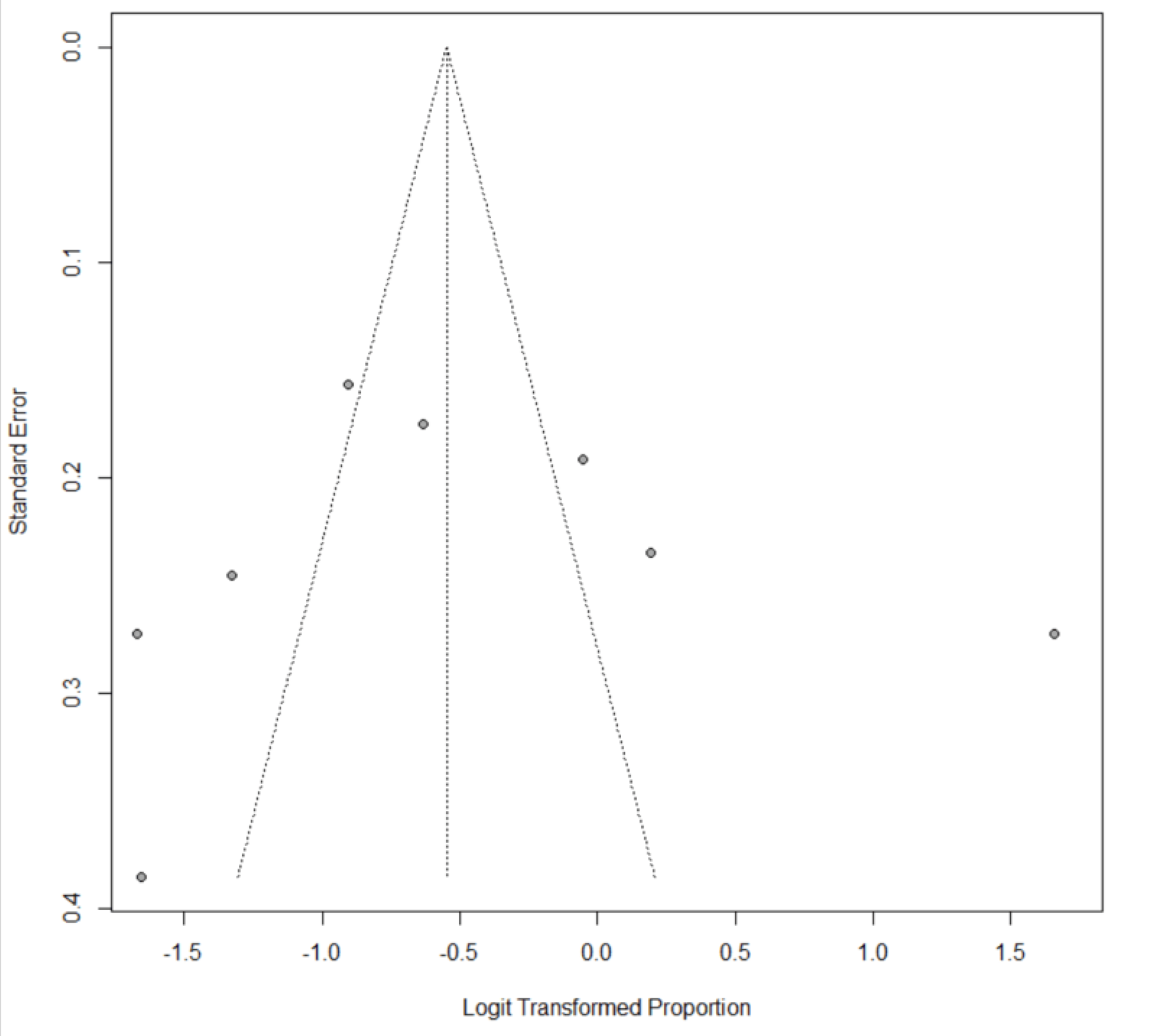

S

2.7 Figure Funnel plot regarding the proportion of obesity among patients with stroke

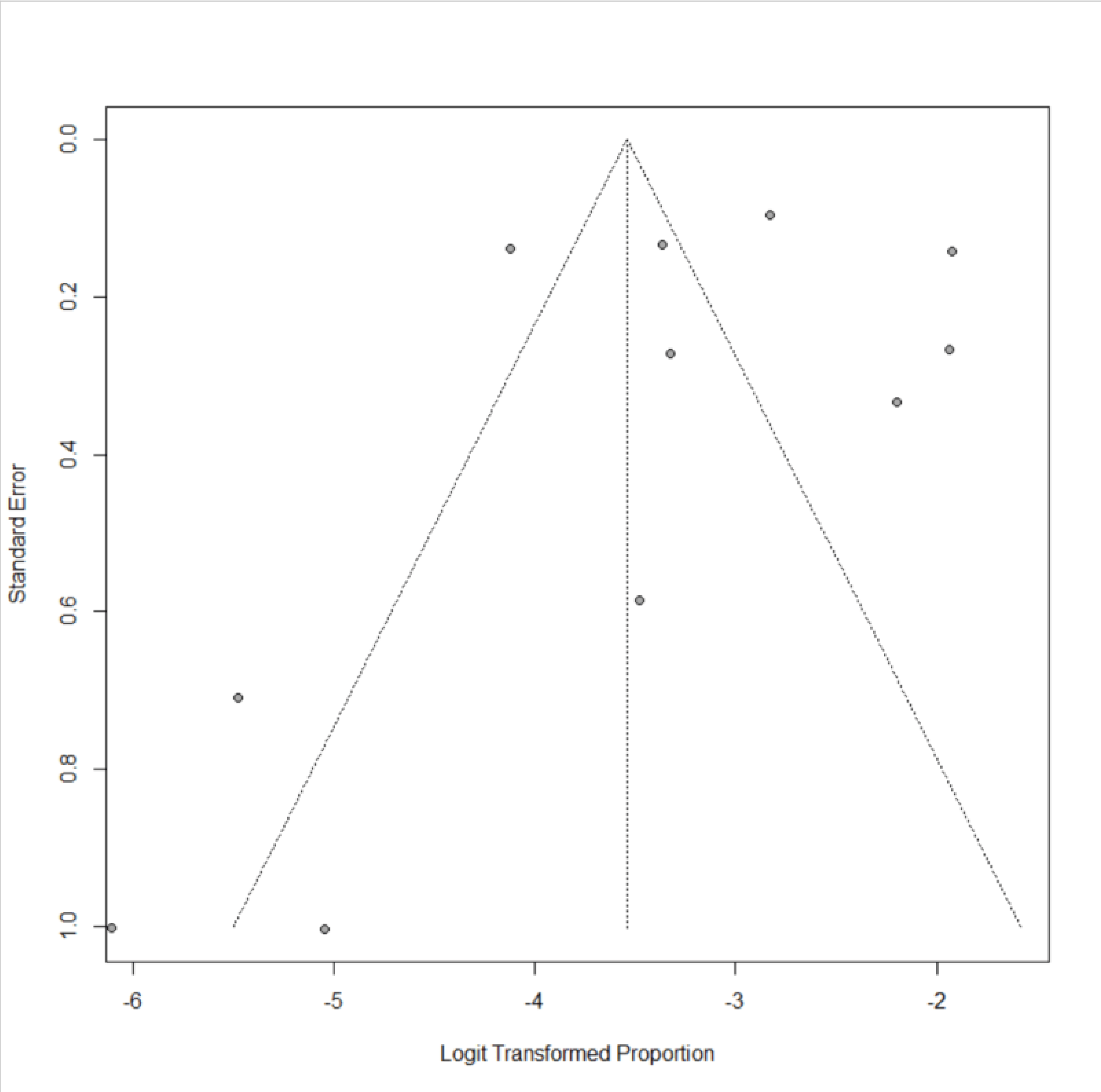

S 2.8 Figure Funnel plot regarding the proportion of rheumatic/ valvular heart disease among patients with stroke

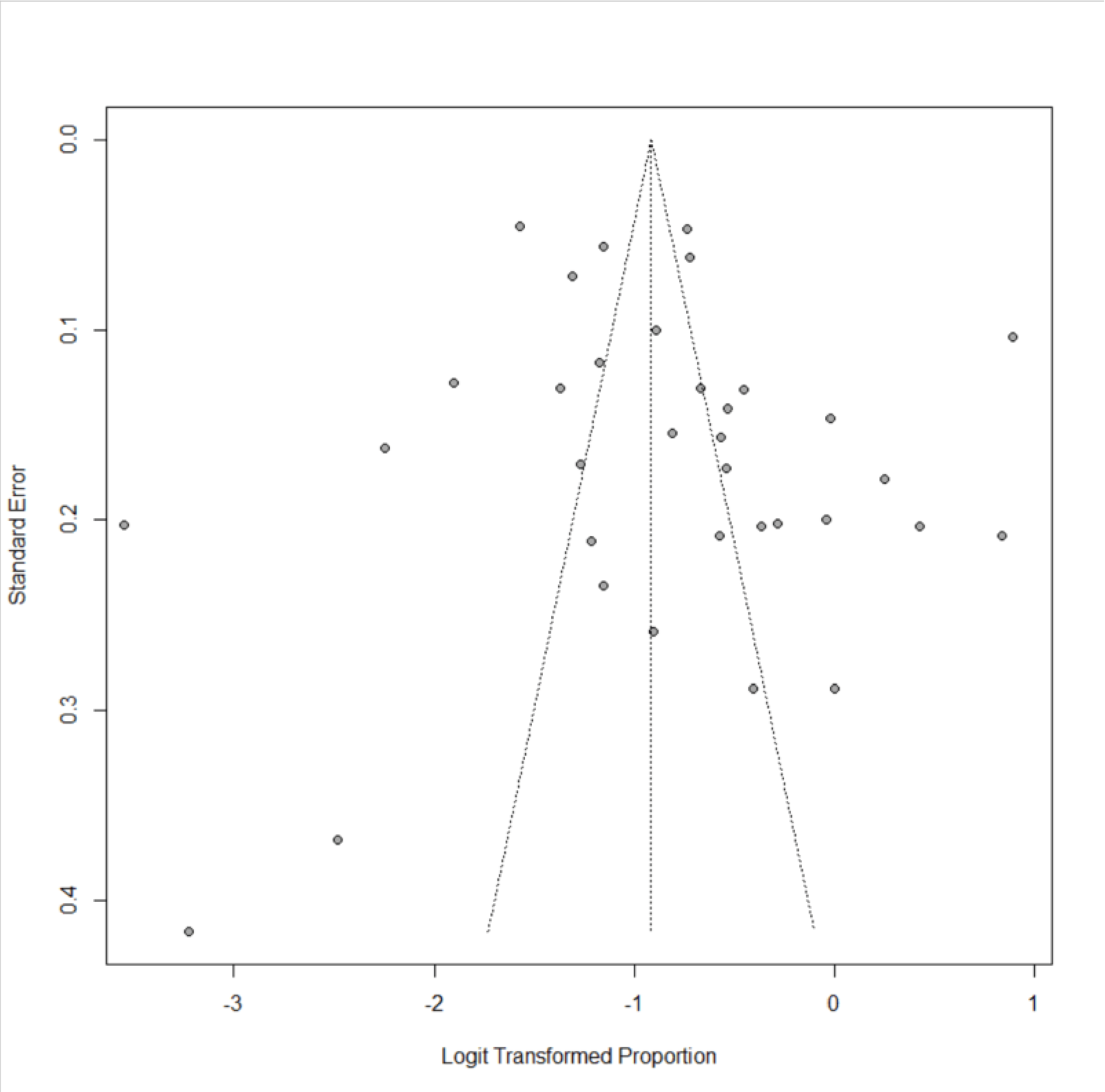

S 2.9 Figure Funnel plot regarding the proportion tobacco use among patients with stroke
